# Virus-like particles and enterovirus antigen found in the brainstem neurons of Parkinson’s disease

**DOI:** 10.12688/f1000research.13626.2

**Published:** 2018-05-02

**Authors:** Robert R. Dourmashkin, Sherman A. McCall, Neil Dourmashkin, Matthew J. Hannah

**Affiliations:** 1 Visiting Research Fellow, Virus Reference Dept., National Infection Service, Public Health England, London, NW9 5EQ, UK; 2 Molecular Pathology, Armed Forces Institute of Pathology, Washington, DC, 20306, USA; 3 Acacia Consulting Sàrl, Luxembourg, L-1244, Luxembourg; 4 Virus Reference Department, National Infection Service, Public Health England, London, NW9 5EQ, UK

**Keywords:** Virus-like particles, Parkinson’s disease, electron microscopy, immunohistochemistry

## Abstract

**Background:** In a previous study on encephalitis lethargica, we identified a virus related to enterovirus in autopsy brain material. Transmission electron microscopy (TEM), immunohistochemistry (IHC) and molecular analysis were employed.  Our present objective was to investigate, using a similar approach, as to whether virus-like particles (VLP) and enterovirus antigen are present in Parkinson’s disease (PD) brainstem neurons.

**Methods:** Fixed tissue from autopsy specimens of late onset PD and control brainstem tissue were received for study. The brain tissue was processed for TEM and IHC according to previous published methods.

**Results:**  We observed VLP in the brainstem neurons of all the cases of PD that were examined.  In the neurons’ cytoplasm there were many virus factories consisting of VLP and endoplasmic reticulum membranes. In some neurons, the virus factories contained incomplete VLP. Complete VLP in some neurons’ virus factories had an average diameter of 31 nm, larger than control brain ribosomes. In the nuclei, there were VLP with an average diameter of 40 nm. In cases of human poliomyelitis, there were cytoplasmic virus factories and intranuclear virus particles similar to those observed in PD. On preparing PD brain sections for IHC there was positive staining using anti-poliovirus antibody and anti-coxsackie antibody. This result was statistically significant.

**Conclusions:** We present evidence for an enterovirus infection in PD.  For future studies, virus isolation and molecular analysis are suggested.

## Introduction

### History

Following our study on encephalitis lethargica ^1^, we were struck by the similarilities of the clinical course of encephalitis lethargica and Parkinson’s disease (PD), leading us to study the latter disease. We have applied the methodology used to characterise encephalitis lethargica by means of TEM of fixed brain tissues and IHC. Our aim is to identify and characterise virus particles and viral products in PD brain.

PD is widespread, especially among the elderly. It was called ‘the shaking palsy’ by Parkinson in 1817 ^2^, and it remains a common cause of debilitation and death. Two hundred years later, we present evidence suggesting an enterovirus infection is present in the brainstem in PD.

Following the pandemic of encephalitis lethargica during World War 1, many of the survivors of the acute phase of the disease developed a syndrome similar to PD, but in which the patients became somnolent. They developed tremor and oculogyric crises during which their eyes rolled uncontrollably ^3^. This syndrome was termed ‘post-encephalitic parkinsonism’ and more popularly in England ‘the sleepy sickness’. It was very common and often conflated with PD. By the end of the 20 ^th^ century every patient who suffered from post-encephalitic parkinsonism had died, whereas PD continued to occur.

### Virology

A number of previous studies have proposed a virus etiology for PD. A review by Jang *et al*. ^4^ discussed the neurological sequelae of infection by influenza virus, as well as that of other viruses known to induce parkinsonism, including Coxsackie, Japanese encephalitis B, St. Louis, West Nile and HIV viruses. Jang *et al*. in an *in vivo* study ^5^ showed that a pathogenic influenza virus can enter the central nervous system causing neuroinflammation and induce neurodegeneration. Zhou *et al.* ^6^ in a literature review suggested that viruses may be associated with neurodegenerative diseases, including Alzheimer’s disease, PD and multiple sclerosis. Duvoisin ^7^ reported in a review the evidence for association of PD with infectious diseases. Hawkes *et al.* ^8^ proposed a dual hit theory for the pathogenesis of infection in PD, in which channels for virus infection pass through the nasal mucosa and the intestinal epithelium. Svensson *et al.* ^9^ and Liu *et al.* ^10^ found a reduced incidence of PD in patients who underwent total vagotomy, suggesting a pathway for infection via the vagus nerve. Gibbs and Gajdusek ^11^ carried out experiments designed to isolate a virus in PD using *in vitro* cell cultures co-cultivated with PD brain tissue. Direct inoculation of PD brain tissue to non-human primates was also carried out. No evidence of PD developed in the inoculated animals and no evidence was found of virus replication in the cell cultures. Wetmur *et al.* ^12^ did not detect nucleic acids complementary to herpes simplex type 1 DNA or influenza A/NWS RNA in the brain of patients with sporadic PD. Elizan *et al.* ^13^ reported that tests were negative for arbovirus antibody using sera from cases of encephalitis lethargica and PD, and in another study ^14^ Elizan *et al.* searched for antibodies to herpes simplex virus 1, measles and rubella in the serum and cerebral spinal fluid of post-encephalitic parkinsonism patients with negative results. Schwartz and Elizan ^15^ did not detect virus particles by means of transmission electron microscopy (TEM) in PD brain tissue nor in cell cultures co-cultivated with PD brain tissue. The possible involvement of enterovirus in PD was not considered in these studies ^11– 15^.

### Transmission experiments

Immature dopamine neurons grafted to PD patients showed that Lewy bodies developed in the grafts ^16^. Jucker and Walker ^17^ found that misfolded α-synuclein can develop disease propagating properties. Human gut microbiota from PD patients induced enhanced motor dysfunction in mice ^18^, suggesting that gut microbiota play a part in the pathogenesis of PD.

### Genetic studies

Davie, in a review of PD ^19^, reported that the LRRK 2 gene (PARK 8) is the most common genetic cause of familial (early onset) PD. The frequency of LRRK 2 mutations in patients with a family history of PD is 5–7%. The heterozygous mutation, 2877510 G>A is the most commonly described, accounting for the majority of familial cases and up to 1.6% of cases of sporadic PD. Mok *et al.* ^20^ found that, in a combined analysis of genome-wide association data of 9,387 cases of PD, eight cases were reported with deletions at the 22q1.2 chromosome site. Age of onset of PD of the patients carrying these deletions was lower (median age, 37 years) than those who did not carry the deletions (median age, 61 years). In the present study, all the PD cases are from the late age of onset.

### Pathology

Pathological studies on the incidence of Lewy bodies in the brain were carried out by Herva and Spillantini ^21^ and Barker and Williams-Grey ^22^, who showed that Lewy bodies were found in 6–8% of autopsies of patients with no clinical evidence of brain disease. The finding of Lewy bodies is the histopathological hallmark of PD; they carry an accumulation of α-synuclein. Barker and Williams-Grey ^22^ reviewed the literature defining α-synuclein as the core protein of Lewy bodies. They are present in PD, in dementia with Lewy bodies, and in multiple system atrophy. Josephs and Parisi ^23^ found that brain tissue from post-encephalitic parkinsonism cases did not carry α-synuclein protein, thus differentiating encephalitis lethargica and the subsequent syndrome, post-encephalitic parkinsonism, from PD.

### Drug induced parkinsonism

Drug induced parkinsonism was described as an acute/subacute syndrome in which tremor is infrequent compared to PD. Most cases of drug induced parkinsonism were caused by both classic and second generation neuroleptics and calcium channel blockers. Resolution by 75% of drug induced parkinsonism occurred six months after withdrawal of the drug ^24^. Thalidomide may worsen existing PD ^25^.

## Methods

### Transmission electron microscopy

Brain tissue from 14 sporadic cases of PD and seven control cases was examined by TEM in order to identify virus-like particles (VLP) in the neurons of the PD brainstem. None of the control cases had poliomyelitis. The method used for TEM of autopsy brain that had been fixed and embedded in paraffin blocks for histology has been previously reported ^1^. Fifty nm sections were cut on a Reichart ultramicrotome, stained with 1% uranyl acetate, then stained with Reynold’s lead citrate, and examined in a JEM1400 TEM (JEOL UK) at Public Health England, Colindale, UK. Images were acquired with an AMT XR60 digital camera (Deben Ltd, UK). VLP in the digital images of cytoplasmic virus factories of PD cases and ribosomes in the cytoplasm of control cases were measured using the measurement facility of the AMT image acquisition software (Deben Ltd. UK). Calliper measurements were also made of each of 30 particles in magnified paper prints of each image ( Table 1). Biased selection of particles for measurement was avoided by measuring all the clearly imaged particles in each print.

**Table 1.** Cytoplasmic particles in two controls (ribosomes) and two Parkinson’s disease (PD) cases (VLP) were measured. 30 particles were measured for each case. The identification of the images is indicated by their author’s TEM serial number. The images used for measurement in Table 1 are illustrated in Figure 3, Figure 4, Figure 14 and Figure 15.

	Control, case no. 10.09013	Control, case no. 12.09003	PD, case no. 14.09.027	PD, case no. 5D.08.17
Figure no.	14	15	4	3
Mean (nm)	19.5	24.2	31.0	30.7
Standard deviation (nm)	4.1	3.4	9.3	5.2

Clinical reports of the PD cases received from the Armed Forces Institute of Pathology (USA) and the John Radcliffe Hospital (UK) stated that Lewy bodies were found by the examining neuropathologists in the brainstem neurons of all the PD cases studied, indicating that PD was present at the time of death.

### Immunohistochemistry

IHC testing was carried out by exactly the same technique described previously ^1^. Rabbit polyclonal anti-polio antiserum raised against polio types 1, 2, and 3, and goat anti-coxsackie polyclonal antiserum raised against a coxsackie virus isolate were donated by the Department of Virology, National Institute for Biological Standards and Control, Potters Bar, UK.

***Method for preparing the polyclonal antibodies***

The inoculation of one rabbit with Poliovirus type 1 for the production of polyclonal antibody:

Virus/adjuvant injected subcutaneously into loose skin, 0.25ml into 4 sites [total 1ml]. Poliovirus Sabin type 1 (10 ^10^ pfu/ml) 1:5 dilution.

*1 rabbit for each virus receives:*

Inoculation 1:

0.5ml virus preparation + 0.5ml TiterMax Gold adjuvant, injected subcutaneously into loose skin, 0.25ml into 4 sites [total 1ml].

Inoculation 2:

3 weeks after 1 ^st^ injection: 0.5ml virus preparation + 0.5ml incomplete TiterMax Gold adjuvant, injected subcutaneously into loose skin, 0.25ml into 4 sites [total 1ml].

Inoculation 3:

3 weeks after 2 ^nd^ injection: 0.5ml virus preparation + 0.5ml incomplete TiterMax Gold adjuvant, injected subcutaneously into loose skin, 0.25ml into 4 sites [total 1ml].

 Sample bleed: after 2 weeks

Followed by terminal bleed 13 days later.

TiterMax ^©^ Gold Adjuvant Sigma Aldrich #T2684

All Plastic Luer lock syringes #Z248010

3-way luer lock stopcock #S7521

The polyclonal antisera were absorbed 5x with normal human autopsy brain tissue. Histologic sections of 12 controls and 21 PD cases were stained with rabbit anti-polio polyclonal antibody, and 14 controls and 19 PD cases were stained with goat polyclonal anti-coxsackie antibody. A series of two-fold dilutions of each antibody was used. The sections were then exposed to biotinylated secondary antibody, then treated with Vector preformed avidin:biotin:enzyme complex ^26^. The slides were then rinsed and stained with Vector Nova Red ^26^, cleared and mounted.

Further IHC tests on PD sections and control sections of normal brain tissue were carried out using the following antibodies: mouse monoclonal antibodies to Enterovirus type 71, Enterovirus VP1 convalescent serum, mouse monoclonal antibody to human parvovirus B19, and serum from human parvovirus infection.

## Results

### Transmission electron microscopy

**Figure 1.**
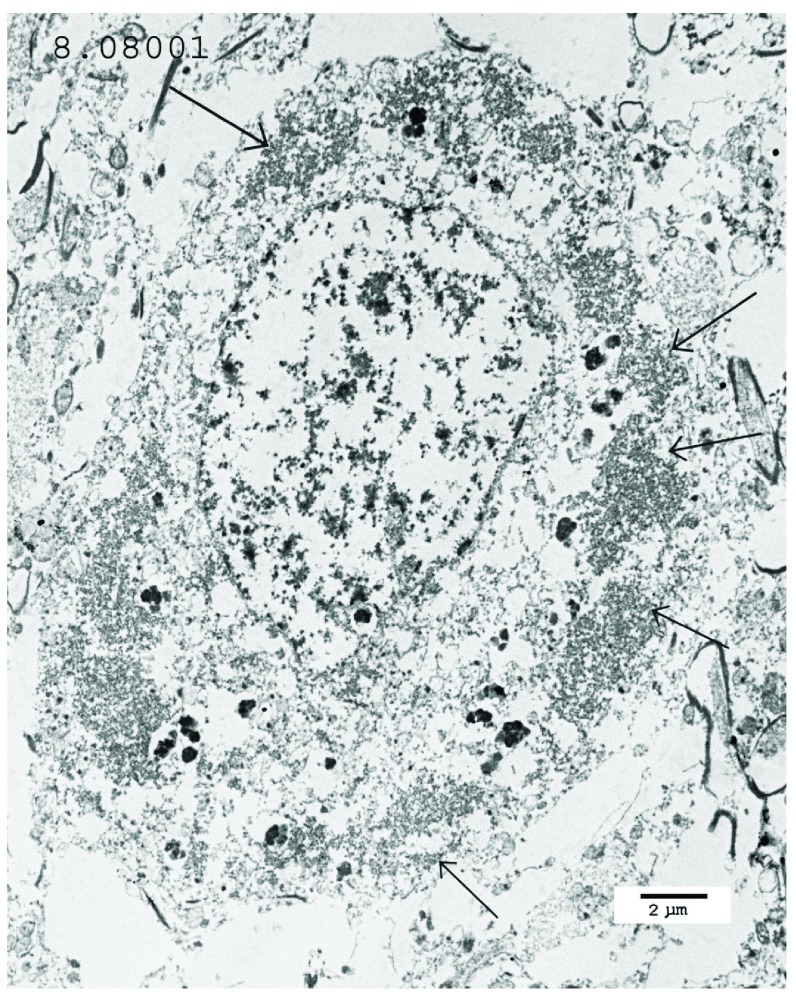
Parkinson’s disease case: TEM image 8.08001,case number 94/1237-6. Low magnification of a neuron in the substanti nigra. The nucleus is almost devoid of chromatin and the emaining chromati is clumped. Many virus factories are in the cytoplasm (arrows). The black granules in the cytoplasm contain melanin.

**Figure 2.**
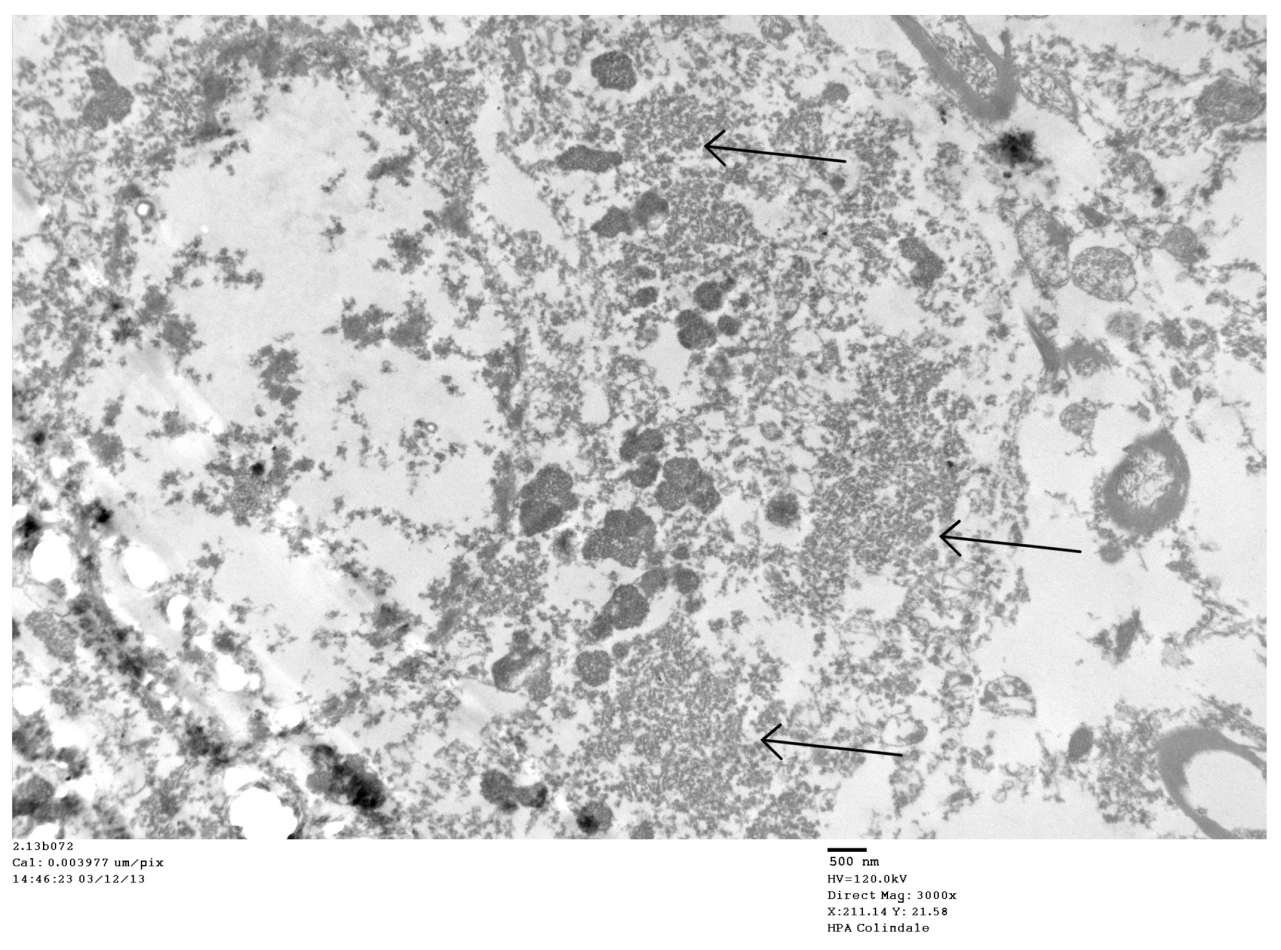
Parkinson’s disease case: TEM image 2.13b072, case number 2844008. Low magnification of a neuron in substantia nigra. Arrows point to virus factories.

**Figure 3.**
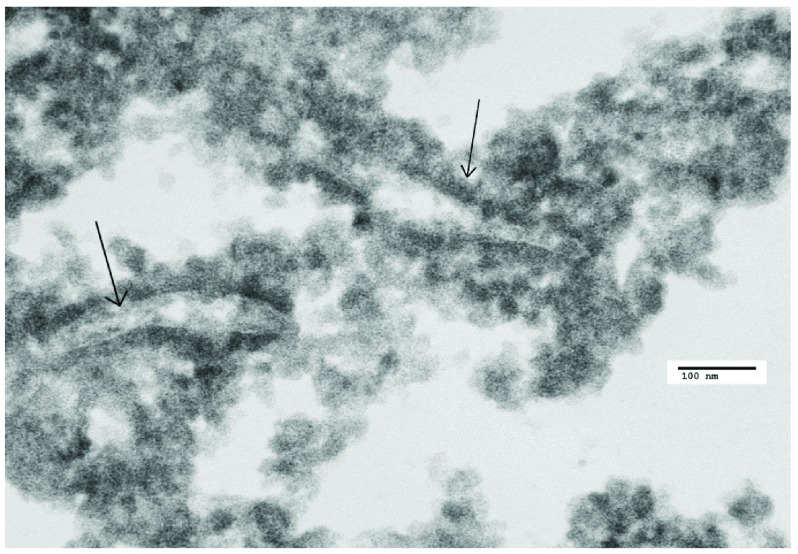
Parkinson’s disease (PD) case: TEM image 5D.08017, case numbers A32-89-19, 2291259. A virus factory in PD. There are endoplasmic reticulum membranes in this image (arrows). There are VLP that are larger than the ribosomes in control Nissl bodies. The mean diameter of the VLP in this image was 31 nm (see Table 1)

**Figure 4.**
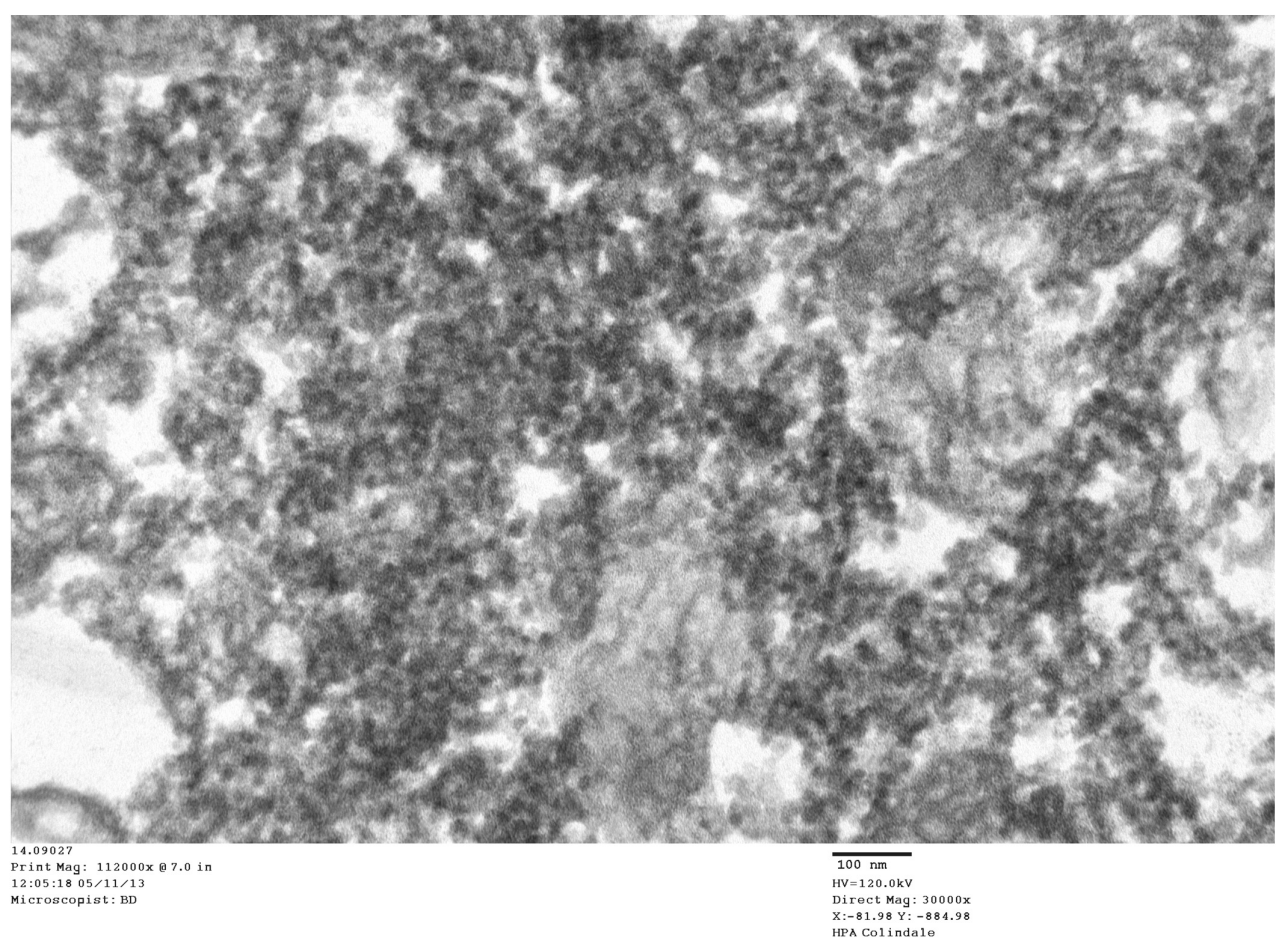
Parkinson’s disease (PD) case: TEM image number 14.9.027, case number 2276564. In the cytoplasm of a PD case, the virus factories consisted of masses of virus-like particles (VLP) in an amorphous matrix. Mean size of VLP: 30 nm (see Table 1).

**Figure 5.**
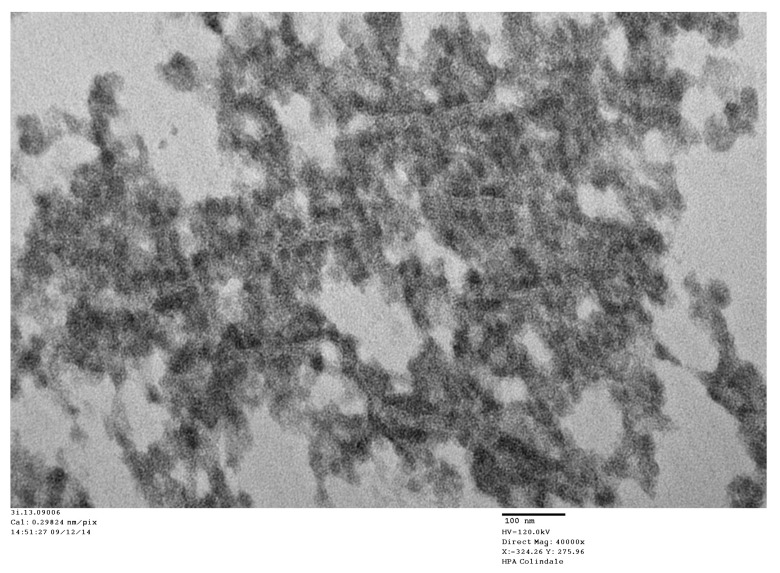
Parkinson’s disease (PD) case: TEM image number 3i.13.09006, case number B2477543. High magnification of a virus factory in a PD neuron showing VLP embedded in virus factory and membrane proliferation.

TEM changes were observed in the brainstem neurons in the PD cases and in the spinal cord motor neurons in the poliomyelitis cases. TEM of PD neurons at low magnification showed advanced apoptosis. There were almost ‘empty’ nuclei with clumped chromatin and multiple cytoplasmic virus factories. Few cytoplasmic organelles remained ( Figure 1 and Figure 2). VLP were found by TEM in the nuclei and cytoplasm in the neurons of all the PD cases studied. The VLP were similar in morphology to the VLP we described in the brain of encephalitis lethargica, which had been confirmed to be a strain of enterovirus by molecular analysis ^1^. The cytoplasmic virus factories in PD neurons consisted of large numbers of VLP interspersed with irregularly shaped endoplasmic reticulum membranes and embedded in virus factory ( Figure 3 and Figure 4). VLP were observed attached to the membranes ( Figure 3). The average measurements of the cytoplasmic VLP in Figure 3 and Figure 4 were both 31 nm ( Table 1). Cytoplasmic virus factories in other PD neurons consisted of incomplete VLP at an early stage of assembly ( Figure 5, see *Discussion*).

**Figure 6.**
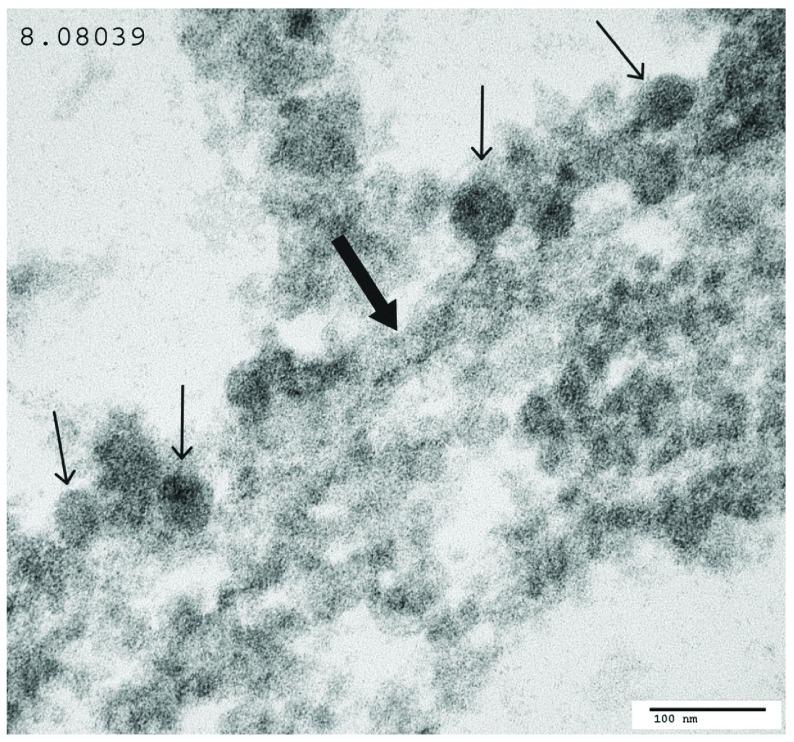
Parkinson’s disease (PD) case: TEM image 8.0839, case number 94/1237-6 (same case as in Figure 1). There are intranuclear VLP lining the internal face of the nuclear membrane of the nucleus. The nuclear membrane is indicated by a thick arrow. VLP are demonstrated by thin arrows.

**Figure 7.**
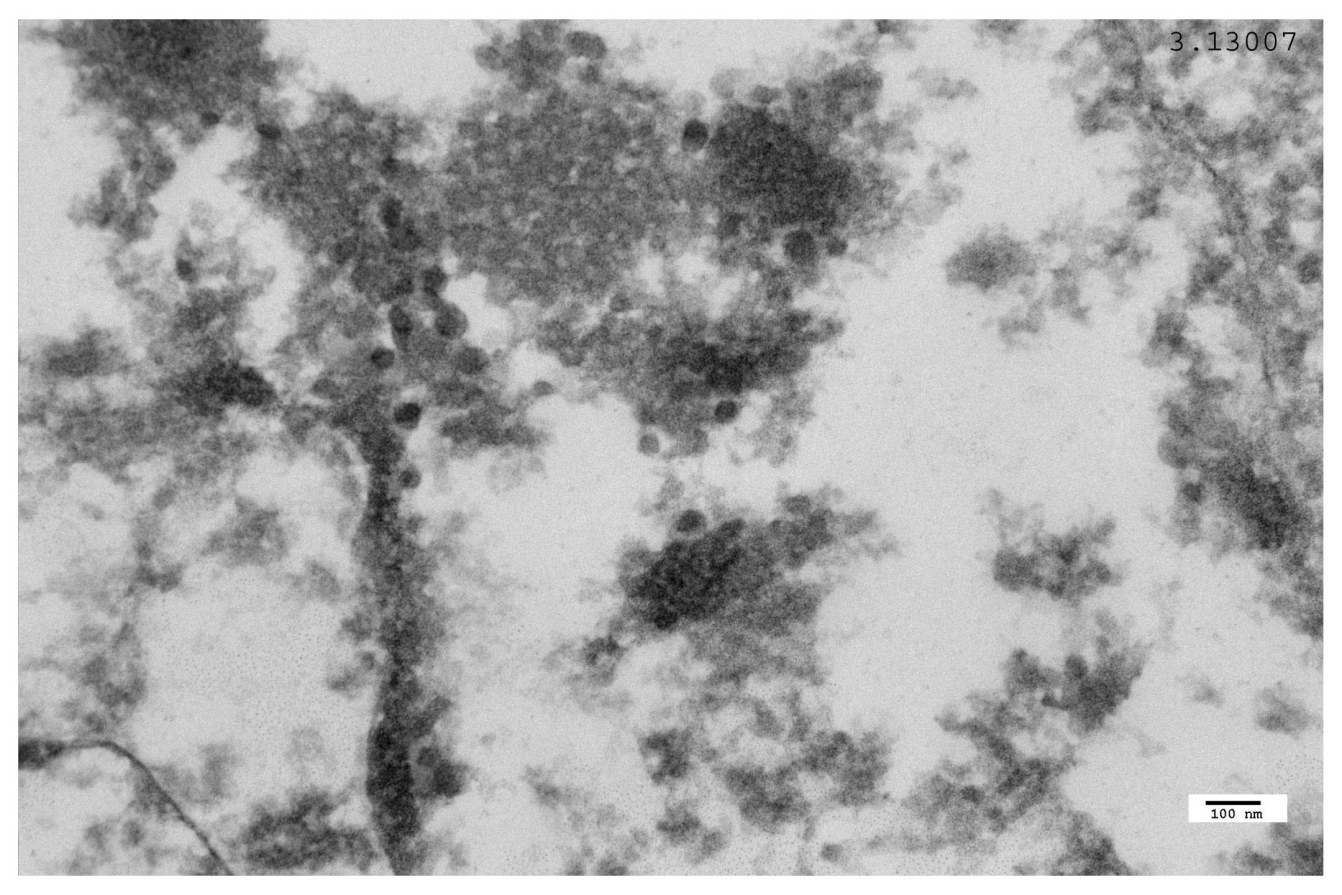
Parkinson’s disease (PD) case: TEM image 3.13.007, case number 2369259 (AFIP). VLP in a PD neuron, close to the internal face of the nuclear membrane.

Intranuclear VLP were found in all 14 cases of PD. The VLP were lined along the internal membrane of neurons and to a lesser degree throughout the nuclei ( Figure 6 and Figure 7). The intranuclear VLP had an average diameter of 40 nm. The shape of the VLP was symmetrical and oval (the latter, the effect of thin sectioning) with clear edges.

**Figure 8.**
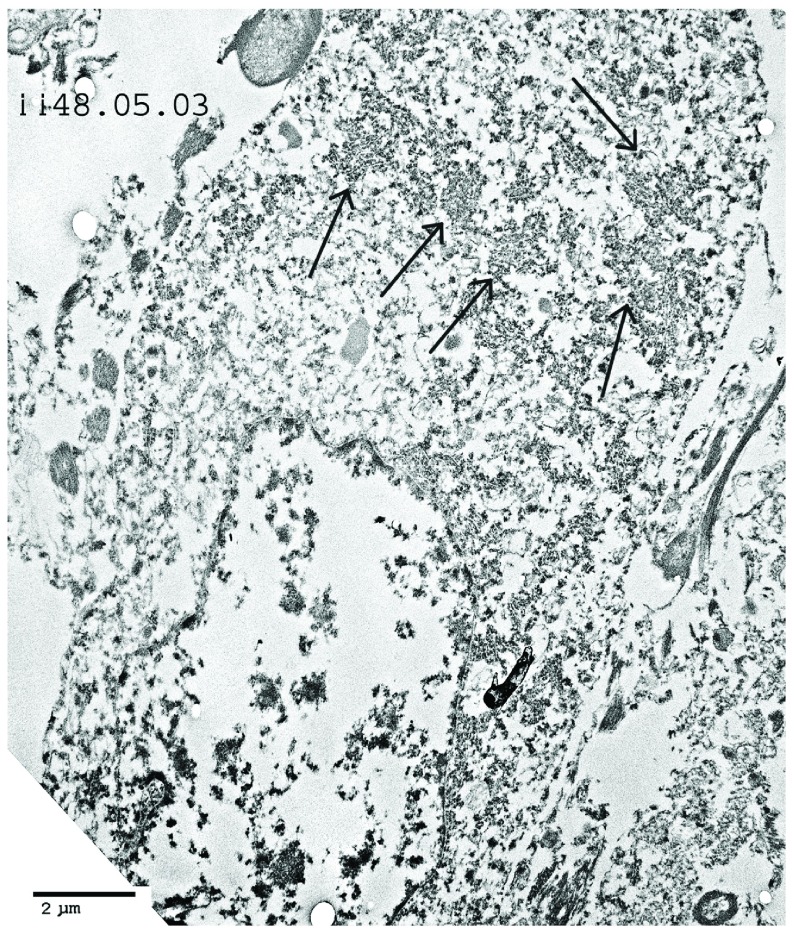
Poliomyelitis case: TEM image ii.48.05.03, case number 702364. Low magnification of a motor neuron. Cytoplasmic virus factories are shown (arrows). The nucleus shows severe apoptosis.

In the cases of human poliomyelitis, motor neurons of the spinal cord showed cytoplasmic virus factories and apoptosis similar to that shown in PD neurons ( Figure 8– Figure 10). Intranuclear virus particles were found that were similar to the VLP in morphology and diameter to those found in the brain of the PD cases ( Figure 11).

**Figure 9.**
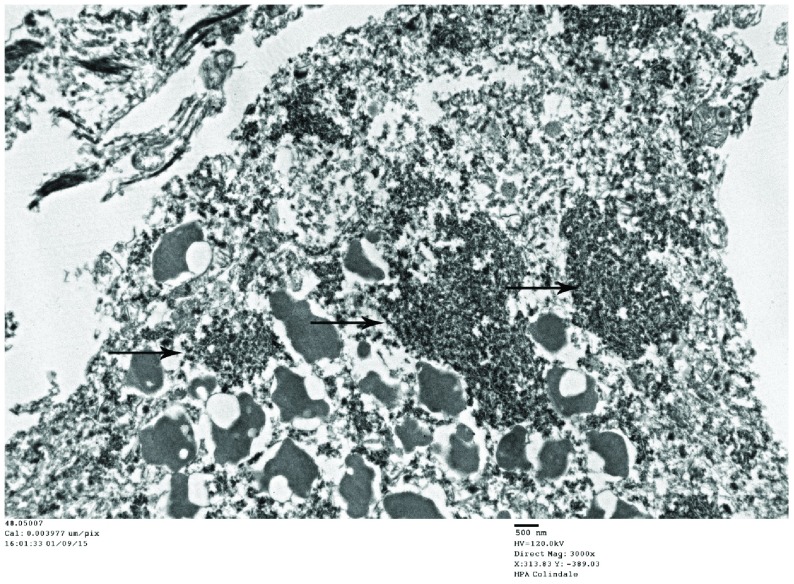
Poliomyelitis case: TEM image ii.48.05.007, case number 702364 (same case as Figure 8). This image shows cytoplasmic virus factories (arrows).

**Figure 10.**
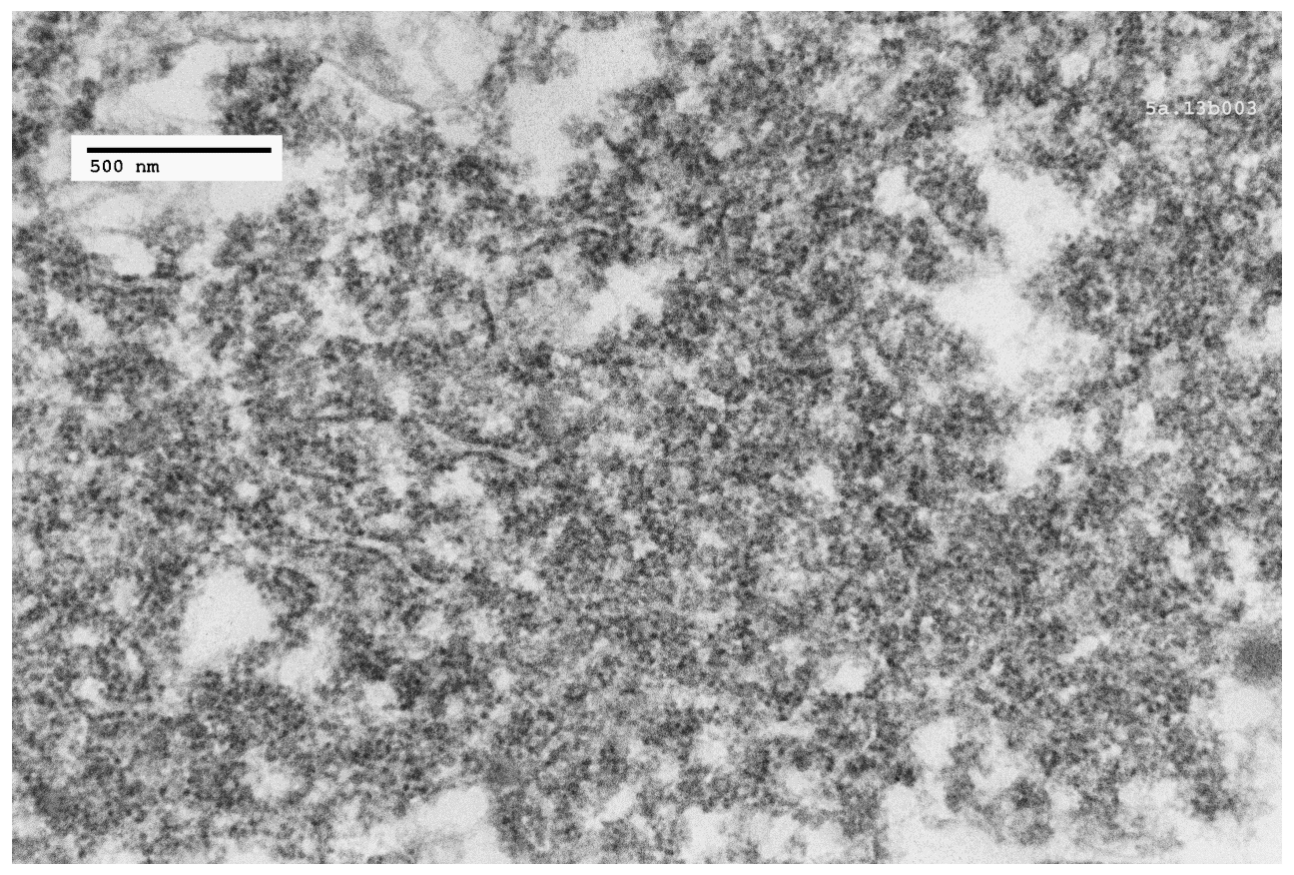
Poliomyelitis case: TEM image 5a.13b003, case number 2134414. A cytoplasmic virus factory, showing strands of endoplasmic reticulum and incomplete virus particles embedded in amorphous matrix.

**Figure 11.**
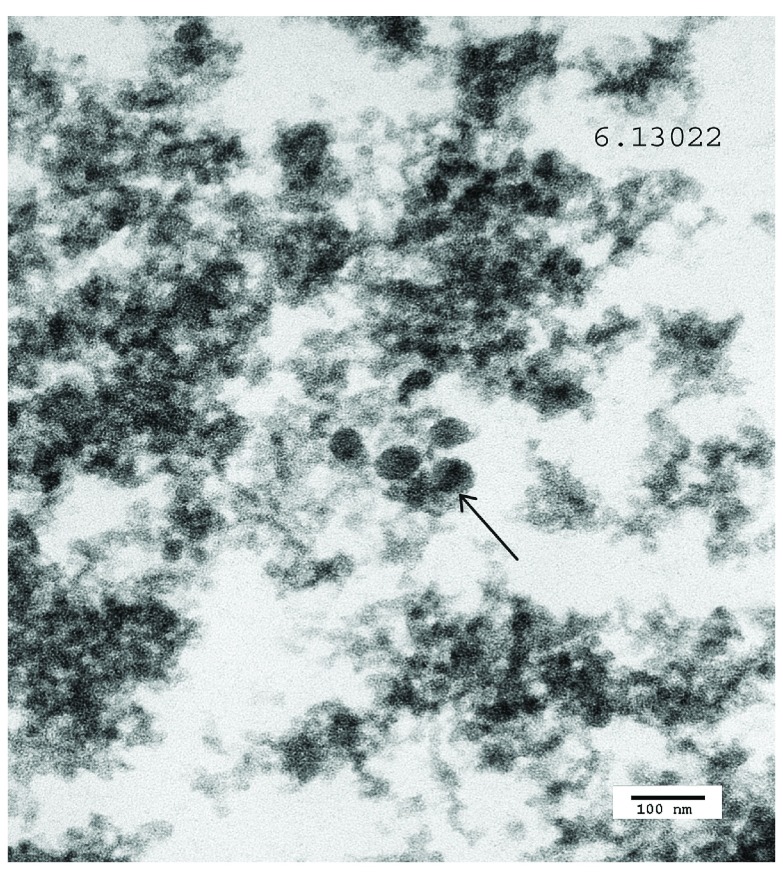
Poliomyelitis case: TEM image 6.13.022, case number 167278. Intranuclear virus particles are shown (arrow).

**Figure 12.**
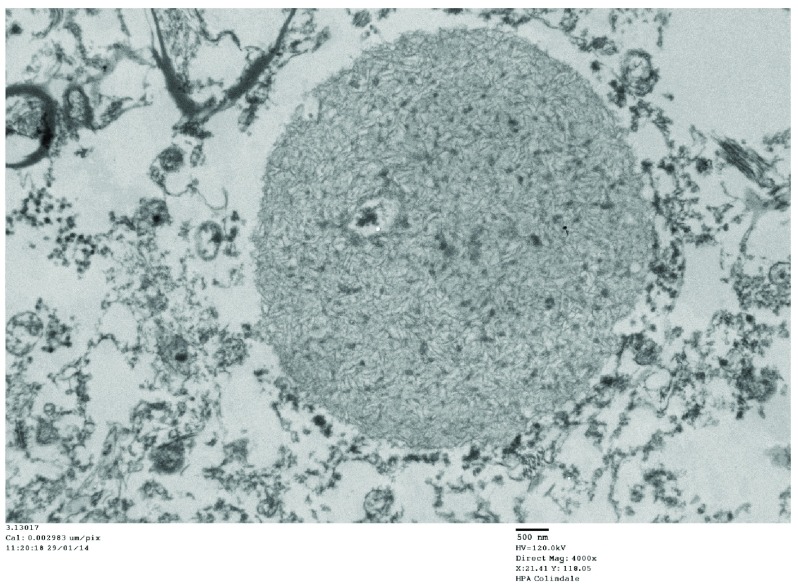
Lewy body in Parkinson’s disease case: TEM image 3.13017, case number 2369259. The cytoplasmic constituents of the cell have degenerated. No VLP were found associated with the Lewy bodies.

A Lewy body is illustrated from a case of PD ( Figure 12). In this image, the nucleus and cytoplasm of the neuron was completely destroyed. There were no VLP associated with the Lewy bodies.

**Figure 13.**
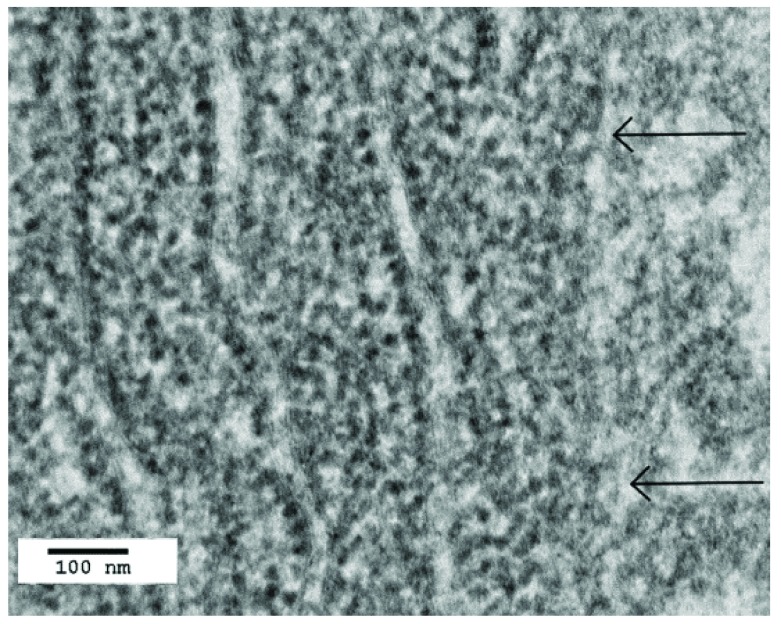
Control case: TEM image 10a.09039, case number 06/112. Control image is presented of cytoplasm of brainstem neuron in a case of myocardial infarction and perforated gastric ulcer. Part of a cytoplasmic Nissl body consisting of endoplasmic reticulum is shown. The nuclear membrane is indicated (arrow).

**Figure 14.**
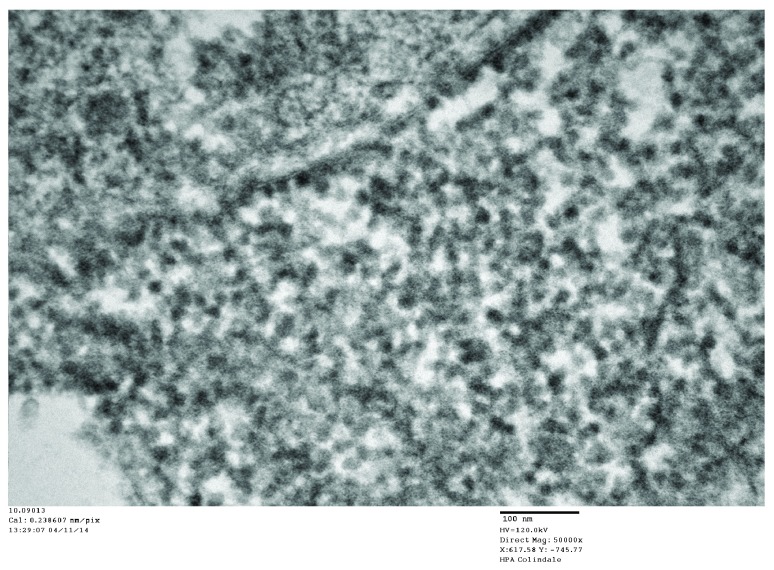
Control case: TEM image 10.0913, case number 06/112. Cytoplasmic ribosomes in a control neuron at high magnification. The mean diameter of the ribosomes is 20 nm (see Table 1).

**Figure 15.**
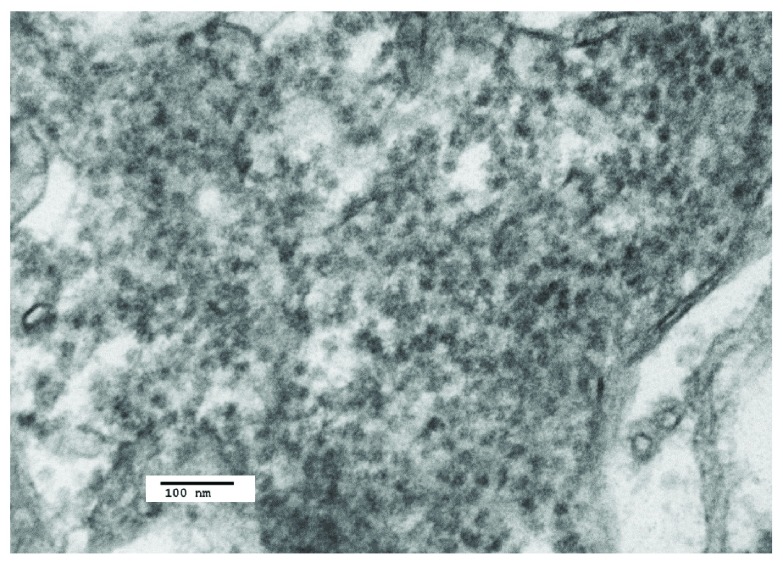
Control case: TEM image 12.09.013, case number 05/152. Image of control brainstem neuron showing cytoplasmic ribosomes (see Table 1).

In the control cases, the neurons showed little apoptosis. Nissl bodies were present consisting of rough endoplasmic reticulum ( Figure 13). Free ribosomes were also found ( Figure 14 and Figure 15). Intranuclear VLP were found in one control case. The neurons in this case showed a moderate degree of apoptosis and lacked cytoplasmic virus factories. The finding of VLP in a control case in this study is in accordance with the observations quoted above, ^21, 22^, in that Lewy bodies were found in the brain of a proportion of control cases. The case reported here could represent a preclinical PD event.

### Immunohistochemistry

IHC staining of PD brain sections showed 13 positive results and 8 negative results using rabbit anti-polio antiserum and 13 positive and 7 negative results using goat anti-coxsackie antiserum; and for control brain sections, there were 10 negative results and 2 positive results for poliovirus antiserum and there were 13 negative results and 1 positive result for coxsackie antiserum. The data for the IHC tests are reported in Table 2 and Table 3. The results were statistically positive for the IHC staining of PD brain tissue as compared with that of control tissue (see *Statistical analysis* section).

**Table 2.** Summary for the TEM and IHC results for the control cases.

Autopsy no.	TEM	Anti-coxsackie antibody	Anti-polio antibody
06/112 (10/09)	-	n.d.	n.d.
06/110 (09/09)	-	-	+
06/126/7 (8/09)	-	-	-
05/152-9 (12/09)	-	-	-
05/25	n.d.	-	-
NP 86/07-4	n.d.	-	-
05/13/75	n.d.	-	-
05/66-28(11/09)	+	-	-
05/01	n.d.	-	-
865183-M	n.d.	-	-
2777945-M (4/14)	-	-	-
8854127-13	n.d.	-	n.d.
883599-11	n.d.	+	+
1311056-17	n.d.	-	-
644255 (38/05)	-	-	n.d.
Totals	6 negative	13 negative	10 negative
	1 positive	1 positive	2 positive
	8 not done	1 not done	3 not done

**Table 3.** Summary of TEM and IHC results for the PD cases.

Autopsy no.	TEM	Anti-coxsackie antibody	Anti-polio antibody
2329162 (4/08)	+	+	+
2291259-6 (5/08)	+	+	+
98/1198-8 (6/08)	+	+	+
98/1167-6 (7/08)	+	-	-
94/1237-6 (8/08)	+	-	-
2297692 (9/08)	+	+	+
2370918 (10/08)	+	+	+
2415938 (11/08, 1/14)	+	+	+
2477543 (13/09)	+	+	-
2276564 (14/09, 14/431)	+	+	+
2344008 (2/13)	+	-	-
2369259 (3/13)	+	-	-
2264415 (9/13)	+	n.d.	n.d.
2419248 (2/14)	+	-	-
A87-2B	n.d.	-	-
2570918	n.d.	n.d.	+
2329162	n.d.	+	+
2870918	n.d.	+	+
2291259	n.d.	+	+
NP31/08-1	n.d.	+	+
2276564	n.d.	+	+
A87-28	n.d.	-	-
Totals	14 positive	13 positive	13 positive
	0 negative	7 negative	8 Negative
	8 not done	2 not done	1 not done

The results were negative for IHC staining of PD tissue using mouse monoclonal anti-enterovirus antibodies as well as monoclonal and polyclonal anti-parvovirus antibody staining. The absence of reaction of enterovirus monoclonal antibodies to PD tissue sections may be attributed to the fact that the solitary epitope to which the antibodies were raised was inappropriate for the staining of PD neurons carrying the putative enterovirus antigen.

**Figure 16.**
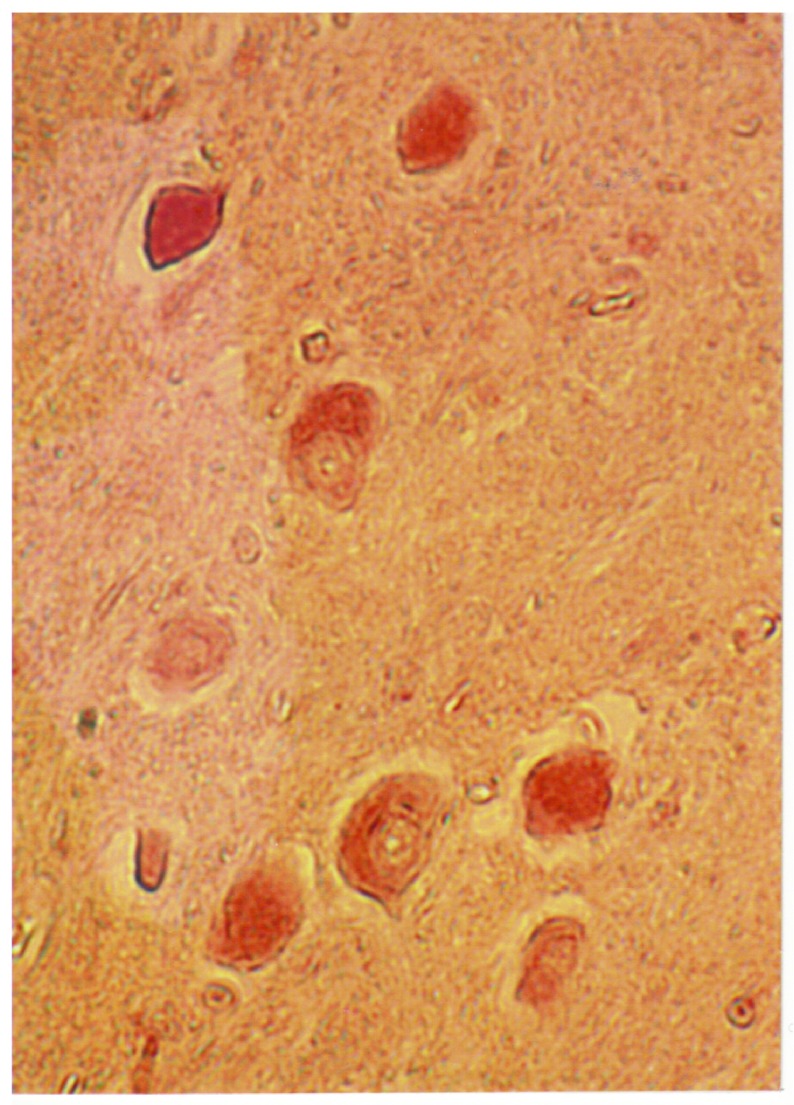
Light microscopy of immunohistochemically stained Parkinson’s disease section: case number 2297692. Illustrated here is an immunohistochemical image of the inferior olive of a neuron using rabbit anti-polio antibody (see *Methods*), showing heavy staining of neurons and lighter staining of surrounding neuropil.

A light microscopy image of an area of the inferior olive of a case of PD is shown that was stained for poliovirus antigen ( Figure 16). There is heavy staining of the neurons and light staining of the surrounding neuropil.

### Statistical analysis

The statistics were calculated using Microsoft Excel 10. The Fisher test results were checked using the ‘R’ statistical package, version 3.3.3. The chi-squared tests were checked against the calculator on http://turner.faculty.swau.edu/mathematics/math241/materials/contablecalc/.

The first null hypothesis, based on the data in Table 1, was that there is no difference between the measured mean size of complete cytoplasmic VLP in each of the PD cases and of cytoplasmic ribosomes in the controls.

A t-test for two independent sample means without assuming equality of variances was used. The resulting probabilities are shown in Table 4. The results are all significant to at least p<.01, and so the null hypothesis is rejected.

**Table 4.** Probabilities that the measurement of complete VLP do not differ from that of control ribosomes. The numbered images correspond to the images that are measured, and also to the figures that are illustrated in this article.

	PD 14.09.027	PD 5D.0817
**Control 10.09013**	2.8 × 10 ^−7^	9.8 × 10 ^−13^
**Control 12.09009**	6.1 × 10 ^−4^	5.9 × 10 ^−7^

The next null hypothesis tested, based on the data for the TEM of PD tissue and of controls was that the positive TEM result is independent of whether the cases are of PD or else controls. Using a chi-squared test for categorical data, the test statistic was calculated at 16.8. With one degree of freedom, the null hypothesis was rejected at the 1% significance level. Using the Fisher exact test, the hypergeometric probability of the results being obtained if the null hypothesis holds is 1.3 × 10 ^-4^. The null hypothesis is thus again rejected.

The third null hypothesis tested was that the observed positive immunohistochemical staining of sections using anti-coxsackie antibody or anti-poliovirus antibody is independent of whether the cases are of PD or else controls. Again using a chi-squared test for categorical data, the test statistic was calculated at 11.4 for anti-coxsackie antibody, which rejects the null hypothesis at the 1% significance level; and 6.3 for anti-poliovirus antibody, which does not reject the null hypothesis at the 1% significance level but does at 5%. Using the Fisher exact test, the hypergeometric probability of the anti-coxsackie antibody results being obtained if the null hypothesis holds is 8.1 × 10 ^-4^, so that the null hypothesis is again rejected at 1% significance level. For the anti-poliovirus antibody, the hypergeometric probability is calculated at 0.014, so the null hypothesis cannot be rejected at the 1% significance level, but is rejected at 5%.

## Discussion and conclusions

In a previous communication, we reported the following TEM finding: the assembly in the nuclei and cytoplasm of polio virus particles and coxsackie virus particles that measured from 20 nm to 40 nm in virus factories of virus infected cell cultures ^1^. The finding of virus particles in the nuclei of cells infected with an RNA virus was a novel observation. Virus factory has been described as a specific intracellular compartment where viral components concentrate by Novoa *et al*. ^27^. Virus factories often include membrane components that are involved in virus replication. This has been demonstrated in our PD study ( Figure 3 and Figure 5). The measurements we made by TEM of poliovirus in infected cells are in contrast to previous reports on the diameter of poliovirus virions using cryo-electron microscopy of purified virus preparations. In the latter studies the diameter of the virions was 30 nm. Larger diameter particles were not reported. This may be due to selection in the purification procedure of the population of virions for study ^28, 29^.

The results of TEM of PD brain are comparable to the TEM finding of virus particles found in cell cultures infected with poliovirus and coxsackie virus ^1^. The VLP found in PD brain are also comparable to the VLP in brain from encephalitis lethargica patients ^1^, and the virus particles found in this study in human spinal cord neurons in cases of human poliomyelitis. We had confirmed the finding of a strain of enterovirus in encephalitis lethargica brain by molecular analysis ^1^. In the present study, the observation that the VLP in many of the cytoplasmic virus factories in the PD cases are larger than the cytoplasmic ribosomes from control cases was confirmed by statistical analysis ( Table 1 and Table 4). In certain PD cases, the cytoplasmic factories consisted of incomplete VLP measuring approximately 20 nm. There was also a membrane component to the virus factories ( Figure 3 and Figure 5). We observed that similar cytoplasmic virus factories were in the human spinal cord neurons infected with polio virus ( Figure 8– Figure 10). These findings suggest that the VLP in PD represent an enterovirus infection. Intranuclear VLP, measuring approximately 40 nm, were observed in the nuclei of motor neurons in cases of poliomyelitis ( Figure 11).

By analogy to recent studies on Alzheimer’s disease ^14, 15^, we suggest that an enterovirus infection in PD may act as a seed for the replication of misfolded α-synuclein protein in addition to the direct cytopathic effect of a virus infection on neurons.

The results of ICH employing polyclonal antisera against coxsackie and polio viruses provide further support for the presence of enterovirus antigen/particles in PD brain. Of 20 samples analysed with coxsackie antiserum 13 were positive while only 1 of 14 controls was labelled. Interestingly 12 of the PD samples that were positive with the coxsackie antiserum were also labelled with the poliovirus antiserum. Previous serological studies ^30, 31^ demonstrated shared epitopes across different enterovirus groups and thus our findings are in keeping with these earlier observations. 

Previous studies did not consider the possibility of enterovirus infection in PD in experiments conducted to isolate virus ^11– 15^. We propose that experiments to isolate enterovirus from PD brain be carried out using PD tissue co-cultivated with cell cultures that carry membrane receptors for enteroviruses. Sequence analysis may be carried out using either virus isolated by cell culture or directly from PD brain tissue using an enterovirus sequence primer. The partial nucleotide sequence of encephalitis lethargica may be employed ^1^.

## Ethical statement

Study of brain specimens had been cleared for ethical agreement by the National Research Ethics Committee for Oxfordshire UK, rec no.: 07/H0606/85. The brain samples from human autopsy material were obtained from the pathology collections of the John Radcliffe Hospital, Oxford UK, and from the former Armed Forces Institute of Pathology, Washington DC, USA. These institutions approved the use of the tissue for research, and they were satisfied that no further ethical approval was required. In the case of the material from the UK, the principal author holds the “Release of Tissue Disclaimer” from the Thomas Willis Oxford Collection, at the Neuropathology Department John Radcliffe Hospital, Oxford.

## Data availability

The data referenced by this article are under copyright with the following copyright statement: Copyright: © 2018 Dourmashkin RR et al.

Data associated with the article are available under the terms of the Creative Commons Zero "No rights reserved" data waiver (CC0 1.0 Public domain dedication). 

Table 2 and Table 3 report the detailed IHC results for IHC tests.

The TEM images presented here are listed in the legends by the author’s TEM serial number followed by the donor’s autopsy number. Copies of the images are stored at the Public Health England electron microscopy laboratory. They may be freely obtained by contacting Dr. Matthew Hannah at PHE.
