# Supplementary material for: Virus-like particles and enterovirus antigen found in the brainstem neurons of Parkinson’s disease
Source: F1000Res. 2018 May 2;7:302. Originally published 2018 Mar 9. [Version 2] doi: 10.12688/f1000research.13626.2 (PMC5968367; doi:10.12688/f1000research.13626.2)
Supplement: Peer review history

# Peer review: Reviewer response for version 2

**DOI:** 10.5256/f1000research.16066.r34094

**Published:** 2018-05-24

**Authors:** Michael M. Frank

**Affiliations:** 1 Duke University Medical Center, Durham, NC, USA

I have now read the paper by Dourmashkin *et al.* Dr. Dourmashkin is a highly qualified electron microscopist with decades of experience. The difference he finds between patient samples and controls is impressive and may lead to treatments that include attempts to prevent viral replication in patients early in disease development.  The paper is inclusive but I was unclear whether any blinded studies were performed.  Were their blinded samples given to the authors and could they identify patient vs. control sample?  Perhaps I missed this in the paper but of course the question is of great interest.

 I approve the paper for publication but do believe that the ability to predict disease vs control material is of great importance.

I confirm that I have read this submission and believe that I have an appropriate level of expertise to confirm that it is of an acceptable scientific standard.

# Peer review: Reviewer response for version 1

**DOI:** 10.5256/f1000research.14803.r31751

**Published:** 2018-04-03

**Authors:** Doris Bucher

**Affiliations:** 1 Department of Microbiology and Immunology, New York Medical College, Valhalla, NY, USA

Comments/minor revisions

- Ref. 16 refers to Treatment of children with human cadaver-derived growth hormone (hGH) contaminated with prions resulted in iatrogenic transmission of CreutzfeldtJakob disease (iCJD).  I was unable to find any reference to ‘immature dopamine neurons grafted to PD patients” and Lewy bodies in Ref. 16.  Need correct reference or further explanation.
- Fig. 4 - would be helpful to use arrows to indicate several VLPs which were used in measurements.
- Fig. 7 - need arrow - VLP In a PD neuron, close to the internal face of the nuclear membrane.
- Re:  Immunohistochemistry Methods:  Was antiserum raised against polio types 1, 2, and 3 or…only poliovirus type 1?
- Fig. 11 - How were these VLP particles identified as intranuclear?  Are smaller particles also seen - i.e. the cluster of particles in the quadrant directly above the putative VLP?
- Fig. 12 - what are the particles seen inside the Lewy body?
- Fig. 16 - Shows only IHC for rabbit anti-polio.  Would be useful to show an example of IHC for anti-coxsackie.
- Table 3 - IHC for anti-coxsackie and anti-polio - nearly all dual positives, only one is specific for coxsackie only.Is this result expected?

I confirm that I have read this submission and believe that I have an appropriate level of expertise to confirm that it is of an acceptable scientific standard.

# Peer review: Reviewer response for version 1

**DOI:** 10.5256/f1000research.14803.r31767

**Published:** 2018-03-21

**Authors:** Steven Patterson

**Affiliations:** 1 Centre for Immunology and vaccinology (CIV), Imperial College London, London, UK

**General comments**

 Ultrastructural and immunohistochemical analysis of brain tissue from Parkinson’s disease patients could provide important data on the pathogenesis of the disease. Electron microscopy of autopsy tissue, particularly after formalin fixation and embedding for light microscopy, is challenging as preservation is far from optimal.  Despite this difficulty the authors have provided persuasive evidence of cytoplasmic structures that may be viral. However, although the authors have convincingly shown differences between Parkinson’s and control brain tissue I feel they should be more cautious in their rather definitive interpretation that there are enterovirus structures antigenically related to polio and coxsachie virus present. In addition I think the discussion should be expanded as indicated below.

**Major specific comments**

1. The ICH showed labelling for both coxsachie and polio virus antigen. What evidence is there in the literature that these viruses have common antigens which may explain their findings? In their studies was either pre-immune or non-immune serum used as a control? Does binding of the coxsachie antiserum block subsequent labelling with the polio virus antiserum and vice versa?
2. The micrographs of the cytoplasmic virus factories in the Parkinson’s tissue are persuasive of enterovirus infection, particularly in view of the similarity to cytoplasmic structures in polio brain tissue and the absence in controls. However, the presence of nuclear virus particles would not be expected of an enterovirus infection. This observation should be more extensively discussed. In addition the extensive early em studies in the 1960s by authors such as Dales (Dales *et al *1965 Virology **26, **389)   should be cited.
3. Is it possible to use haemotoxylin to stain nuclei in the IHC to confirm or not the presence of nuclear viral antigen?   
4. Table 3 summarises the em and IHC data showing that for 13 samples there was both em and IHC. All 13 were positive for virus factories by em but 8/13 and 7/13 were positive for staining with coxsachie and poliovirus antiserum respectively. Given that it may be expected that IHC is more sensitive since a larger area of tissue may be examined the authors should comment on the difference between the em and IHC findings.

**Minor comments** 

1. I presume that the tissue was fixed in formalin, this should be stated in methods
2. For the reader it would be helpful if a micrograph could be shown in which both ribosomes and VLPs are indicated
3. Figure 7 legend states that an arrow indicates the nuclear membrane, I cannot see an arrow on this micrograph.
4. There appears to be a core in one of the nuclear particles shown in figure 11, indicated by the arrow, can the authors comment.

I confirm that I have read this submission and believe that I have an appropriate level of expertise to confirm that it is of an acceptable scientific standard, however I have significant reservations, as outlined above.

# Response

**Published:** 2018-05-08

**Authors:** Steven Patterson

**Affiliations:** Imperial College London, UK

The amendments made by the authors have satisfied my concerns.

 Steven Patterson
